# A Guide to Bioinformatics for Immunologists

**DOI:** 10.3389/fimmu.2013.00416

**Published:** 2013-12-04

**Authors:** Fiona J. Whelan, Nicholas V. L. Yap, Michael G. Surette, G. Brian Golding, Dawn M. E. Bowdish

**Affiliations:** ^1^Department of Biochemistry and Biomedical Sciences, McMaster University, Hamilton, ON, Canada; ^2^Department of Biology, McMaster University, Hamilton, ON, Canada; ^3^Department of Pathology and Molecular Medicine, McMaster University, Hamilton, ON, Canada

**Keywords:** bioinformatics, immunology, sequence alignments, single-nucleotide polymorphisms, transcriptional profiling, scavenger receptor

## Abstract

Bioinformatics includes a suite of methods, which are cheap, approachable, and many of which are easily accessible without any sort of specialized bioinformatic training. Yet, despite this, bioinformatic tools are under-utilized by immunologists. Herein, we review a representative set of publicly available, easy-to-use bioinformatic tools using our own research on an under-annotated human gene, SCARA3, as an example. SCARA3 shares an evolutionary relationship with the class A scavenger receptors, but preliminary research showed that it was divergent enough that its function remained unclear. In our quest for more information about this gene – did it share gene sequence similarities to other scavenger receptors? Did it contain conserved protein domains? Where was it expressed in the human body? – we discovered the power and informative potential of publicly available bioinformatic tools designed for the novice in mind, which allowed us to hypothesize on the regulation, structure, and function of this protein. We argue that these tools are largely applicable to many facets of immunology research.

## Introduction

Although public perception indicates that bioinformatics is a relatively new discipline borne out of the “omics” age, bioinformatics is more than just “data crunching” and, in some form, has been around longer than our understanding of how DNA translates into protein. The term “bioinformatics” was coined in 1970 by Hogeweg and Hesper to mean “the study of informatic processes in biotic systems” (1). In this sense, the interdisciplinary approach characteristic of bioinformatic’s combination of information science, mathematics, and biology is not a new venture. Even before the term was ever used, Erwin Schrodinger, recognizable for his thought experiments and developments in quantum mechanics (2), gave a series of lectures in war-time Ireland entitled *What is Life?* (3), encouraging many classically trained physicists and chemists, including Francis Crick and Rosalind Franklin, to turn their interests toward biology. These new recruits became some of the first interdisciplinary scientists. Since then, it has been used for a broad range of applications, including the Human Genome Project (4), the discovery of new drugs (5), and further elucidation of Darwin’s Tree of Life (6).

Just as bioinformatics can be applied to the study of human genetics and evolution, it can also be used to inform immunology research. This combination of immunology and computational biology is sometimes referred to as “immunomics” or “computational immunology.” Bioinformatic techniques have been used to model how major histocompatibility complex (MHC) heterozygosity affects one’s interaction with bacteria (7) and the influenza virus (8), how host stress affects the pathogenicity of *Pseudomonas aeruginosa* in the human gut (9), and why the frequency of staphylococcal-induced toxic stress response is low even though infections by these bacteria are high (10). While some of these investigations require a user to have extensive knowledge of computational science, increasingly, bioinformatic tools are equipped with intuitive graphical user interfaces and so are more accessible to those without such a background. Many powerful and informative results can be generated with an Internet connection and a DNA sequence of interest. The plethora of publicly available, easy-to-use bioinformatic tools that investigate nucleotide or protein sequences, can provide information about potential post-translational modifications, predict protein structure and gene expression, and document genetic variation within a population, species, or kingdom. Within minutes, information can be generated to guide *in vitro* experiments, which can save the typical bench scientist both time and resources.

This review uses recent examples of our own quest to seek out information on a potential member of the class A scavenger receptor family, SCARA3, via publicly available bioinformatic tools. The scavenger receptors are a family of proteins required for host defense and phagocytosis of senescent cells and modified proteins (11). Although SCARA3 is a member of this family, there is very little information on its structure or function. Through an example of our bioinformatic analyses of the SCARA3 gene, this review aims to explain how approachable and accessible bioinformatic tools can be used to obtain sequence and structural information, gene expression patterns, genetic variation across human populations and, most importantly, to generate informed hypotheses that can be tested bench-side.

## Sequence Analysis

### Acquiring a FASTA sequence from a public online database

The FASTA file format was originally described by William R. Pearson as part of his 1990 bioinformatic software package of the same name (12). Since this time, it has become the *de facto* file format for most, if not all, bioinformatic sequence analyses. Simply put, this format is a description of a sequence preceded by a greater-than (“ >”) symbol, followed by the sequence in the standard IUPAC nucleotide or protein code.

An accurately annotated and appropriately formatted sequence of the gene(s) of interest is a prerequisite of many bioinformatic techniques. Since 2007, the National Center for Biotechnology Information (NCBI) has made the nucleotide sequences of more than 260,000 organisms accessible through its publicly available database, GenBank (13). GenBank’s global coverage of sequence data is ensured by daily exchanges of information with the European Molecular Biology Laboratory’s (EMBL) Nucleotide Sequence Database, and the DNA Data Bank of Japan (DDBJ) (13). The information stored in GenBank is made accessible through Entrez, NCBI’s comprehensive search engine (13). Users of Entrez have the option of searching within specific databases, such as nucleotide and protein sequences, Expressed Sequence Tags (ESTs), and macromolecular structures (14).

One such database is Entrez Gene, which provides gene-centered information (15). Entrez Gene includes only those gene records corresponding to genomes which have been fully sequenced or to genes that have active research groups associated with them (15); searches of this or other curated databases avoid poor search results. Additionally, because some annotations in complete genomes are quite suspect, the use of Entrez Gene prevents the use of inappropriately annotated or low quality sequences. Searching this database provides useful information such as the “*Genomic regions, transcripts, and products*” section, which is helpful in visualizing the exonic structure and chromosomal orientation of a gene. The “*Bibliography*” section summarizes peer-reviewed articles in which the gene is at the forefront. Additionally, a multiple sequence alignment of the gene of interest to known homologs can be generated by choosing the “*Homology*” section under “*General gene information*”; this may be of interest to those conducting cross-species or evolutionary studies.

**Figure 1:**
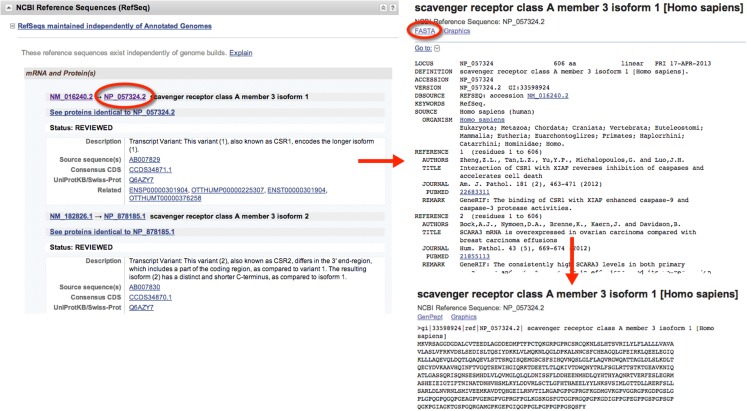
**Retrieval of nucleic acid and protein FASTA formatted sequences from an Entrez Gene search**. Upon searching for and selecting the *Homo sapiens* SCARA3 gene, a variety of information can be retrieved including identifiers for the Ensembl, Mendelian Inheritance of Man (MIM), and Human Protein Reference Database, in addition to information about the genomic context of the gene. From the “*NCBI Reference Sequences (RefSeq)*” section, the most up-to-date and thoroughly curated FASTA formatted sequences may be obtained. Sequences with Accession Identifiers beginning with NM or XM are mRNA and NP or XP are protein. Multiple RefSeq entries may be present in the case of gene isoforms. Selecting the NP_057324.2 Accession Identifier, information concerning the SCARA3 isoform 1, protein is displayed, including links to publications involving this protein. By selecting “FASTA” at the top of the page, the FASTA formatted sequence is provided, which includes the reference number, species, and name. This sequence is suitable for input into most online bioinformatic tools.

When gathering sequence data, the user should refer to the section entitled “*NCBI Reference Sequences (RefSeq)*” (Figure 1). Using RefSeqs is important because these sequences meet a stringent standard set by NCBI, including the assurance that supporting evidence for the gene is available (16). Here, at least one set of mRNA and protein sequences will be displayed; isoforms of a given protein are displayed with multiple entries.

Although we have chosen to use the NCBI’s Entrez platform in this example it should be noted that there are other equally appropriate databases available. Although it is beyond the scope of this review to describe them in detail, Table 1 provides an overview.

**Table 1:** **Public databases containing DNA, mRNA and protein sequences**.

Acronym	Name	Hosted by	URL	Features	Reference
GenBank	GenBank	National Center for Biotechnology Information	http://www.ncbi.nlm.nih.gov/genbank/	An annotated collection of all publicly available DNA sequences (EST, gene and transcript sequences and unannotated single read sequences from genome sequencing projects)	Benson et al. (13)
EMBL- BANK	EMBL Nucleotide Sequence Database	European Molecular Biology Laboratory (EMBL)	http://www.ebi.ac.uk/embl/	A collection of DNA and RNA sequences submitted by researchers, genome sequencing projects, and patent applications. In addition to querying individual genes, whole genomes may be browsed	Kulikova (56)
DDBJ	DNA Data Bank of Japan	DNA Data Bank of Japan	http://www.ddbj.nig.ac.jp/	A collection of nucleotide sequences where sequences of recently sequenced genomes are particularly well represented	Miyazaki (57)
UCSC	UCSC Genome Bioinformatics site	Genome Bioinformatics Group at the University of California Santa Cruz	http://genome.ucsc.edu/	Contains reference sequences and working draft assemblies for a large collection of genomes. Source of sequences for genomes that have not been comprehensively sequenced and annotated (e.g., Neadertal)	Kent et al. (58)

### Predicting post-translational modifications

Post-translational modifications of a protein can include phosphorylation, glycosylation, ubiquitination, methylation, and lipidation amongst many others. Post-translational modification may change the function, cellular localization, or abundance of a protein. Just as understanding protein domains and genomic context can inform the function of a protein, understanding how a protein is post-translationally modified may provide important clues regarding function. For example, signal transduction mediated by the immunoreceptor tyrosine-based activation motif (ITAM) of the T-cell receptor, requires the dual phosphorylation of two of its tyrosine residues [reviewed in Ref. (17)]. Predictions as to which of the many possible post-translational modifications are statistically likely in a given protein may explain cellular localization patterns, regulation of protein abundance, and indicate whether the protein contains specific signaling properties.

**Figure 2:**
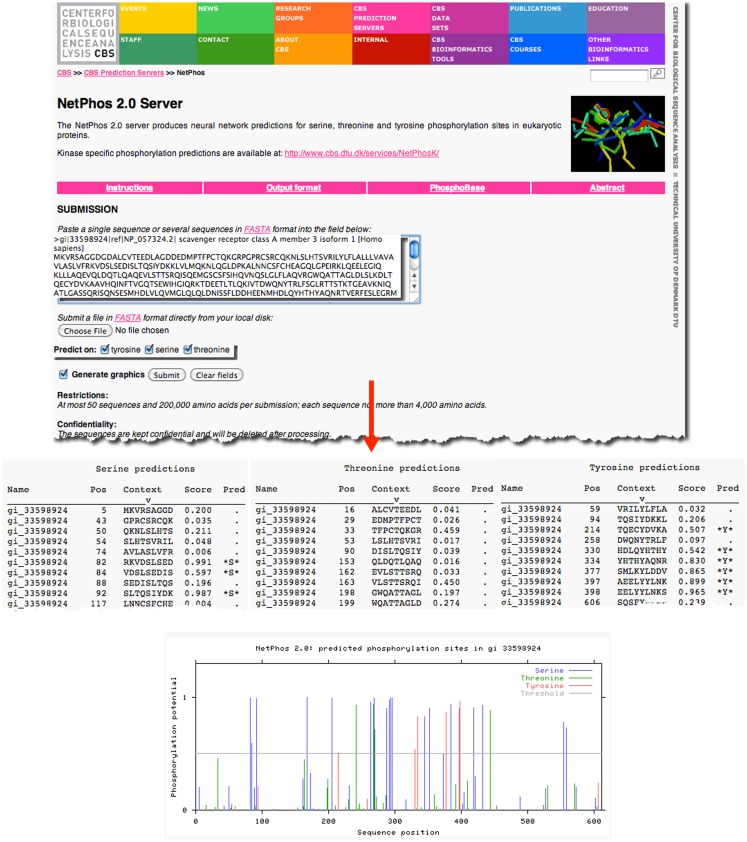
**Prediction of post-translational modifications in SCARA3**. The FASTA formatted sequence of SCARA3 from *Homo sapiens* was entered into the NetPhos 2.0 Server to predict serine (S), threonine (T), and tyrosine (Y) residues that may be phosphorylated. Each instance of these residues and surrounding sequences are displayed under the “*Context*” column. Scores above 0.5 are considered to be significant and those residues are highlighted in the “*Pred*” column with asterisks. The Server also displays the output graphically, including a horizontal line to indicate the 0.5 score threshold. Multiple residues in SCARA3 reach this threshold of significance, and may guide further *in vitro* analysis of this protein.

As an example, previous research has demonstrated that the prototypical member of the class A scavenger receptors, SRAI, has a serine in the cytoplasmic domain of this protein, which, when phosphorylated, is essential for its phagocytic function (18, 19). However, it is not known whether the other members of the class A scavenger receptor family, such as SCARA3, contain similar sites of post-translational modifications. Knowledge of such sites would suggest that SCARA3, like SRAI, is also a phagocytic receptor whose signaling pathways are conserved within this receptor family. The SCARA3 FASTA formatted protein sequence obtained from NCBI was analyzed using the NetPhos 2.0 Server (Figure 2). This tool was built on the knowledge that the 7- to 12-amino acids neighboring a phosphorylated residue tend to have a specified composition in order to be recognized by specific kinases and phosphatases (20). Using this information, NetPhos predicts sites of phosphorylation in a protein sequence. In the case of SCARA3, multiple sites were identified over the threshold probability value defined by the software to be serine (S)-, threonine (T)-, or tyrosine (Y)-phosphorylated (Figure 2), indicating that even though these residues differ from those identified in SRAI, SCARA3 may possess similar functionality.

In addition to NetPhos, there are many post-translational modification prediction tools publically available which require the sole input of a protein sequence. A representative collection of these tools is summarized in Table 2.

**Table 2:** **A representative collection of bioinformatic tools for post-translational modification (PTM) prediction**.

Name	Hosted by	PTM predicted	URL/Reference
NetCGlyc 1.0 Server	Center for Biological Sequence Analysis (CBS)	C-mannosylation sites in mammalian proteins	http://genome.cbs.dtu.dk/services/NetCGlyc/; Julenius (59)
NMT	The Research Institute of Molecular Pathology (IMP) Bioinformatics Group	The MYR predictor for prediction of N-terminal N-myristoylation of proteins	http://mendel.imp.univie.ac.at/myristate/SUPLpredictor.htm
PrePS: Prenylation Prediction Suite	The Research Institute of Molecular Pathology (IMP) Bioinformatics Group	Predicts whether a protein is prenylated	http://mendel.imp.ac.at/PrePS/; Maurer-Stroh and Eisenhaber (60)
NetPhos 2.0 Server	Center for Biological Sequence Analysis (CBS)	Predictions of phosphorylation sites on serine, threonine, and tyrosine residues	http://genome.cbs.dtu.dk/services/NetPhos/; Blom et al. (20)
The Sulfinator	ExPASy Bioinformatics Resource Portal	Prediction of tyrosine sulfation sites	http://web.expasy.org/sulfinator/; Monigatti et al. (61)
SUMOplot Analysis tool	Abgent	Predict the probability of sumoylation sites within a protein sequence	http://www.abgent.com/tools/
ProP 1.0 Server	Center for Biological Sequence Analysis (CBS)	Predicts arginine and lysine propeptide cleavage sites	http://genome.cbs.dtu.dk/services/ProP/; Duckert et al. (62)
UBPred	Indiana University, Columbia University, University of California, San Diego, CA, USA	Predicts protein ubiquitination sites	http://www.ubpred.org/; Radivojac et al. (63)

*There are many publically available PTM prediction tools that require only the input of a protein sequence. This table outlines a representative subset that are available as online tools*.

### Identifying conserved motifs

Some regions of a gene are more susceptible to the accumulation of mutational change over evolutionary time than others and protection from change is largely due to the biological importance of such a region (21). Highly conserved regions have generally been demonstrated to encode for areas essential for a protein’s expression or function where even slight changes would threaten the organism’s survival. In contrast, in other areas of a protein, neutral mutations that do not affect protein function may accumulate over time (21). By examining areas of conservation in a protein of interest across its orthologs (i.e., genes separated by a speciation event; the same gene in different species) and paralogs (i.e., genes separated by a gene duplication event; similar genes in the same species) one can predict regions that are important for expression or function (22).

This is accomplished by performing sequence alignments. An alignment of sequences simply put, is the addition of gaps (represented as “-”s) at variable positions in a set of input sequences in order to maximize the number of similar residues per column in the alignment (22). These alignments come in a variety of forms: first, they can either be “*pairwise*,” involve only two sequences, or “*multiple*,” involve more than two sequences. Second, they can be “*global*,” which means the full length of all sequences are aligned, or “*local*,” indicating that the best alignment is displayed, even if that means only aligning a portion of the inputted sequences to each other (23). The use of pairwise versus multiple sequence alignments depends on how many closely related proteins the user has at their disposal; the more sequences, if they are closely related, will better inform the alignment. However, the choice of local versus global alignments is not as straightforward. The results of local alignments are often more meaningful because the method emphasizes regions of high similarity between sequences (23). These types of alignments are quite informative when comparing divergent protein sequences that are hypothesized to share a specific protein domain. However, often a researcher is interested in comparing full-length sequences of high similarity to each other, in which case a global alignment must be employed.

**Figure 3:**
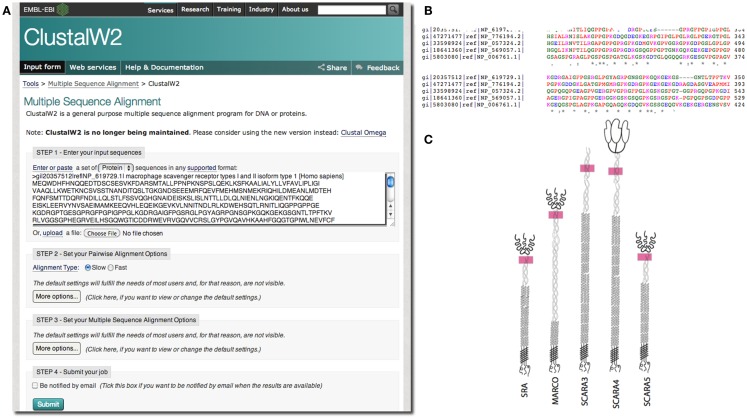
**Use of multiple sequence alignments to discover regions of evolutionary conservation and presumed functionality**. FASTA formatted protein sequences of the scavenger receptors were obtained as described previously for SRAI (NP_619729.1), MARCO (NP_006761.1), SCARA3 (NP_057324.2), SCARA4 (NP_569057.1), and SCARA5 (NP_776194.2) and inputted into the Multiple Sequence Alignment tool, ClustalW2 **(A)**. The sequences were aligned; a portion of the alignment with the highest conservation across all five sequences is shown **(B)**. The user may choose to view colored output, where red represents small, hydrophobic amino acids (AVFPMILW), blue represents acidic amino acids (DE), magenta represents basic amino acids (RK), and green represents STYHCNGQ (hydroxyl, sulfhydryl, amine, and glycine). Coloring allows the viewer to visualize the distribution of charge and hydrophobicity in the protein. In this example, we see that there is an orderly distribution of hydrophobic amino acids (red). The degree of consensus is represented with symbols. (*) Indicates positions which have a single, fully conserved residue; (:) indicates conservation between groups of strongly similar properties; (.) represents conservation between groups of amino acids with weakly similar properties. The fact that all five members of this family share this highly conserved region at locations in these proteins indicated with pink rectangles, **(C)**, and that it is the highest area of conservation within the proteins is strongly suggestive of a conserved function.

In our case, we were interested in the similarities of SCARA3 to the other members of the class A scavenger receptors (its paralogs) that, to date, have been better characterized in terms of biological function and expression. Any similarities between specific regions of SCARA3 and these well-characterized cousins would allow us to hypothesize that these regions perform similar functions in both proteins. As such, we computed a global alignment of the human SCARA3 protein with the other four members of this protein family (Figure 3). A global sequence alignment is used in this case because previous research has suggested that these proteins have evolved in parallel for many millions of years, resulting in some similar biological functions, suggesting that they share areas of similarity across the full lengths of these proteins (11, 24).

European Molecular Biology Laboratory’s European Bioinformatics Institute (EBI) has a set of tools available for both pairwise^1^ and multiple sequence alignments^2^. In the example in Figure 3, we perform a global multiple sequence alignment of the class A scavenger receptor protein sequences from *Homo sapiens* using the ClustalW2 tool (Figure 3A). ClustalW2 was chosen because it is suitable for “medium-length” alignments, which is perfect for analysis of the scavenger receptors, which are approximately 500 base pairs in length. Additionally, ClustalW2 produces a colorful output, which makes it easy to visualize conserved residues and patterns of charge or residue repeats by visual inspection. A portion of the results of this alignment can be visualized in Figure 3B. Notably, this alignment identified an area of conservation at the C-terminal region of the collagenous domain across all five members of the class A scavenger receptors (Figure 3C). This area, consisting of predominantly charged amino acids, has been previously implicated in ligand binding in SRAI (25). Consequently we might predict that this region is a ligand-binding site not only in SRAI, but also in the other four members of this protein family.

Another approach to the identification of conserved motifs, especially useful when no known homologs exist, are specialized tools that examine an input sequence for known domains. An example of such a tool is NCBI’s Conserved Domain Search (CD-search) which compares a user-provided sequence against an NCBI-curated database of known domains (26). These tools do not find the intricacies of sequence alignments but can, however, be very informative.

## Structural Analysis

### Acquiring publically available macromolecular structures

Of course, while clues to a protein’s function can be hidden within its sequence, at the end of the day, it’s the protein’s structure that dictates its function. Because of the ease of DNA and protein sequencing given today’s technologies, there is more sequence data available compared to structural evidence; however, databases with structural information are available. The Protein Data Bank (PDB) is a worldwide collection of macromolecular structures governed by the Research Collaboratory for Structural Bioinformatics (RCSB). This online, searchable database^3^ has come a long way from its meager beginnings as a repository established in 1971 for seven structures, as it is now home to 92104 structures and counting (27). Each experimentally validated entry is assigned a PDB Identifier that can be used to search against the database. Alternatively, information such as the molecule name or author may be used.

**Figure 4:**
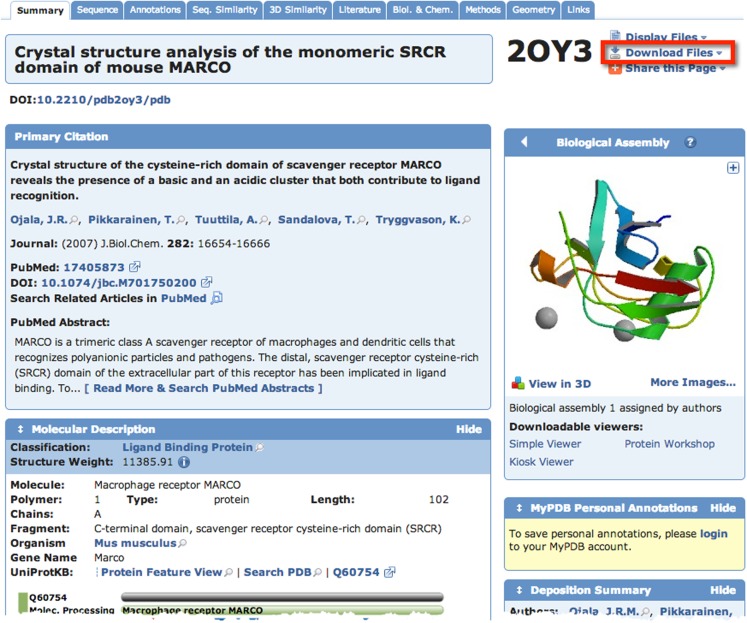
**The Protein Data Bank (PDB) entry for a macromolecular structure of a scavenger receptor**. Because crystal structures of proteins are more difficult to obtain than their protein sequences, the PDB database is less populated than sequence databases such as NCBI’s Entrez. However, PDB is still an excellent resource. Here, an example of the detailed entry for PDB ID 2OY3 is displayed after a search for “MARCO” was performed. Information is displayed such as the primary citation from which this structure was submitted, and a small visualization of the structure. Further, more detailed visualizations can be created easily by the user by downloading the .pdb formatted file from the top right of an entry, and displaying it in software such as UCSF Chimera.

A quick search of PDB with the search term “SCARA3” resulted in no hits. This is unsurprising given that little work has been done with this protein. However, since we know from our sequence analyses that there are regions of homology between SCARA3 and the other receptors, it is worth searching for these proteins as well. A search for “MARCO” revealed a structure (PDB ID: 2OY3) of the SRCR domain of the mouse MARCO protein (Figure 4). The PDB entry for this structure includes information such as the citation to the original publication, the functional classification of this region, its molecular weight, and an exportable macromolecular structure. Structures can be downloaded in a variety of formats, including as a form of coded text saved as a .pdb file or as a static.jpg image. The .pdb file gives the user a chance to interact with the structure by moving it along an axis, coloring based on amino acid type, or calculating potential protein-ligand interaction partners. These types of manipulations can be implemented in freely available software such as UCSF’s Chimera (28) or others summarized in Table 3.

**Table 3:** **Summary of publicly available software for the modeling of macromolecular structures**.

Name	Hosted by	URL	Features	Availability	Reference
UCSF Chimera	Resource for biocomputing, visualization, and informatics at University of California, San Francisco, CA, USA	http://www.cgl.ucsf.edu/chimera/	Allows interactive visualization of macromolecular structures. Along with .pdb files, one can also import density maps, sequence alignments, and trajectories among other information. Python script plugins	For download on all major platforms	Pettersen et al. (28)
BioBlender	Science visulization unit, Consiglio Nazionale Delle Ricerche (CNR)	http://bioblender.eu	Built as an extension of blender, open-source 3D modeling software used for video games and animation, is able to display physical and chemical properties of a protein	For download on all major platforms	Andrei et al. (64)
Jmol	Various	http://jmol.sourceforge.net	Visualization of 3D protein structures in a variety of input formats including .pdb, can measure distances in Å. Great introductory animation at URL	Web applet	(65)

*These software can import .pdb formatted files for viewing and/or manipulation and modeling*.

Unfortunately for our explorations of SCARA3, our previous sequence analyses indicate that the SRCR domain of MARCO –the only current macromolecular structure of a scavenger receptor – is not a region that is shared between these two receptors and, thus, it does not indicate any new information about our protein of interest. As structural prediction technologies improve, and more experiments are conducted, the size of PDB will grow, but even in its current state it is an excellent resource for structural information.

### Protein structural predictions

However, even if an experimentally verified protein structure such as those in PDB does not exist for a protein of interest, predictions as to the potential secondary structure of a protein can still be made based on the primary protein sequence. One common method is the reliance on identifying similar motifs in a protein sequence of interest when compared to a well-studied protein with known function (29). However, use of this method risks the transfer of incorrectly annotated information from protein to protein, thus potentially causing the corruption of genome databases if perpetuated (30). Other methods are based on highly complex algorithmic analyses, which make simplifying assumptions that exchange some accuracy for an algorithmic solution (31). These algorithms take into account certain patterns characteristic of a secondary structure, which tend to be represented in the primary sequence. For example, collagen, the main constituent of connective tissue, is generally encoded as a combination of glycine, proline, hydroxyproline, and hydroxylysine (32). These patterns allow bioinformatic tools to predict certain secondary structures such as collagenous regions from a primary sequence.

Psipred is an excellent example of such a predictive tool. Psipred is an online resource, which combines multiple secondary structure prediction methods into one, easy-to-use web-interface (33). First, psipred generates a sequence profile of the user’s sequence using BLAST, which determines areas of conservation and variation (33). Conserved areas denote areas of functionality, as well as areas that form the core of the protein; whereas, variable regions not responsible for specific folds, or the integrity of the protein structure generally exist on the surface (33). These sequence profiles give this tool its first hints as to the protein’s structure. Subsequently, an algorithmic approach is used to compare those patterns found in the sequence of interest to those identified in other proteins.

**Figure 5:**
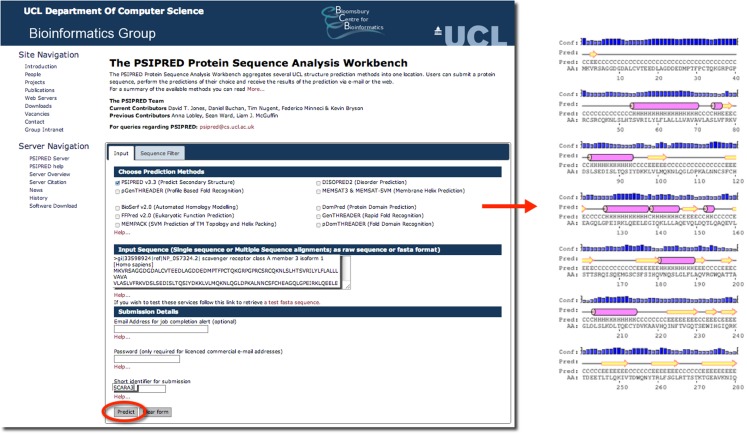
**The use of Psipred for the prediction of the secondary protein structure of SCARA3**. The Psipred tool combines various secondary protein prediction algorithms into one web-interface. Upon inputting the NCBI RefSeq protein sequence of SCARA3, Psipred outputted structural predictions, including the location of α-helices (pink cylinders) and β-sheets (yellow arrows).

The results of inputting the human SCARA3 protein sequence into the online Psipred tool gave us an indication of which segments of the sequence formed α-helices and β-sheets (Figure 5). When we were analyzing the protein sequences of all the scavenger receptors as part of our determination of the evolution of the protein family (24), we were able to build off of this information to discover that some of the predicted α-helix segments were indeed coiled-coil motifs based on the form HxxHcccH where hydrophobic (H) residues were interspersed with other amino acids (x), some of which were more likely to be charged (c) (34, 35). There are a few other tools that work in a similar fashion to Psipred, which we have reviewed in Table 4.

**Table 4:** **Tools for the prediction of secondary structure characteristics**.

Name	Hosted by	URL	Features	Reference
psipred	University College London (UCL) Department of Computer Science	http://bioinf.cs.ucl.ac.uk/psipred/	Uses PSI-BLAST to determine regions of homology which inform their predictions	Jones (33)
JPred	University of Dundee	http://www.compbio.dundee.ac.uk/www-jpred	Takes into account solvent accessibility in its predictions; displays PDB matches if applicable	Cole et al. (66)
CFSSP (Chou and Fasman Secondary Structure Prediction) Server	BioGem.org	http://biogem.org/tool/chou-fasman/	Uses the Chou and Fasman algorithm to predict helices, sheets, turns, and coils	Chou and Fasman (67)

In addition to these general tools, there are others that focus on predicting specific aspects of different types of proteins. The TMHMM Server, for example, focuses on the prediction of transmembrane domains using a statistical model (36). Output from this tool, indicates whether a protein has a transmembrane domain and its predicted location. Additionally, tools such as SignalP focus on the prediction of signal peptide cleavage sites within an amino acid sequence, which can add to the user’s knowledge of a protein’s structure (37).

## Transcriptomics

### Gene expression profiles to answer immunological questions

Studies of global gene expression (“transcriptomics”) using microarrays, RNA sequencing (RNAseq), and other platforms have been a valuable tool for immunologists. Transcriptomics can be used to discover “*gene signatures*” of disease states or to provide mechanistic insight into disease etiology. Because variability within individuals dictate symptoms and disease progression, it is very rare that changes in expression of a single gene will be sufficiently robust for diagnosis; however, combinatorial changes that indicate a common mode of regulation are more robust and allow for the formation of “gene signatures.” For example, an “interferon signature” of gene expression was discovered in lupus when type I interferon inducible genes were found to be elevated in the peripheral blood mononuclear cells (PBMCs) of patients with lupus compared to healthy controls (38). Other notable discoveries in immunology made using transcriptomics include the discovery of the mechanisms of genetic regulation associated with lipopolysaccharide (LPS) tolerance (39), predicting long-term survival from breast and other cancers (40), and studying changes in microbial gene expression over the course of disease (41). As the immunology community’s use of transcriptomic data increases, public repositories such as the NCBI’s Gene Expression Omnibus^4^, EBI’s Gene Expression Atlas^5^, and other specialized sites such as http://www.macrophages.com/ contain a rich amount of data waiting to be mined. These resources include transcriptional profiles of different immunological cell types and activation states in a wide range of organisms. Although there are challenges with comparing microarray data from different platforms and sources (42) the cost savings of reproducing publicly available experiments have increased the appeal of utilizing public resources.

**Figure 6:**
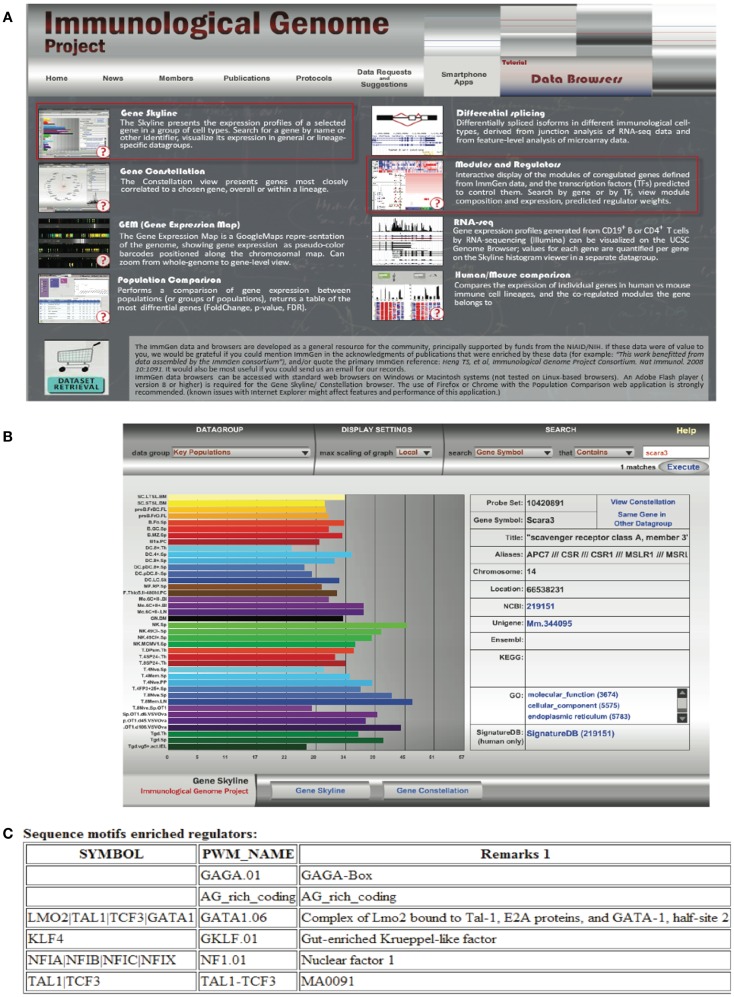
**Querying the Immunological Genome Project (http://immgen.org) for data on expression and transcriptional regulation of SCARA3**. **(A)** The Immunological Genome project has a number of ways to browse the data and visualize patterns of gene expression and transcriptional regulation. **(B)** Using the “Gene Skyline” browser we see that the transcript for SCARA3 is expressed at low levels in most cell types in the database. **(C)** Using the “Modules and Regulators” browser we see that there are four predicted transcription factor binding sites (NF1.01, GATA1.06, GKLF.01, and TAL1-TCF3) and two regulatory regions (GAGA.01, AG_rich_coding) in the promotor of SCARA3.

Transcriptomics has also fed the immunologist’s obsession with characterizing leukocyte subsets and lineage. In some cases, defining cells by their transcriptional profile has proven to be as effective as sorting by flow cytometry (42). These data have inspired researchers to search for the holy grail of transcriptional profiles that characterize subsets of immune cells and are more specific than surface markers. Although this approach has been somewhat successful [e.g., in identifying a novel subset of NK cells; (43), for cell types such as macrophages and dendritic cells that seem to have a more plastic phenotype and ontogeny, the usefulness of this approach has been a subject of debate (44, 45)]. Nonetheless this quest has inspired the creation of the Immunological Genome Project^6^ (46). This consortium of researchers is characterizing the transcriptional profile of immune cells based on rigid sorting and purification profiles, and although these data consist almost entirely of mouse genes in the steady state, it is a valuable resource to the immunology community. In our attempt to learn about SCARA3, we used the “*Gene Skyline*” and “*Modules and Regulators*” tools (Figure 6A) to find that transcripts of SCARA3 are expressed broadly across a wide range of cells at relatively low abundance (Figure 6B). There is no published data describing how SCARA3 is transcriptionally regulated; however, four transcription factor binding sites (NFIA, TAL1, KLF4, and LMO2) and two regulatory regions are predicted to occur in the promotor region of SCARA3 (Figure 6C). The Immgen database allows researchers to glean a considerable amount of data about their gene of interest with very little investment or specialized knowledge.

Although the Immgen database is probably the most user friendly, it is dominated by mouse immune cell subsets. Other resources such as IRIS (Immune response *in silico*) take a similar approach to characterizing the transcriptional profiles of human leukocyte subsets and include different activation states (47).

## Genetic Variation

### Analysis of single-nucleotide polymorphism

The most common type of variation within the human genome are single-nucleotide polymorphisms (SNPs), which occur, on average, every 1200 base pairs (48). SNPs can be non-synonymous or synonymous; non-synonymous SNPs result in a change in the amino acid sequence of the translated protein, while synonymous SNPs do not alter the amino acid composition because of the redundancy of the genetic code.

Single-nucleotide polymorphism analysis of a protein can greatly aid in the understanding of its function as these small alterations can result in substantial changes in the functionality of the protein. For example, a SNP at a receptor’s binding site may alter the original protein such that it would be able to bind a pathogen that it previously was unable to, or, in contrast, may abolish its ability to bind its usual binding partner. In one study, researchers studied differences in SNP frequencies of Mal/TIRAP to explain differences in TLR2 and TLR4 signaling between European and African populations (49). After cloning the two variants, S180L and S180, results indicated that S180L heterozygous individuals had a higher cytokine production level than S180 homozygous individuals (49). Lower allele frequencies of S180L in African and Asian populations might indicate selection occurred after humans migrated from Africa since the variant may have granted added bacterial resistance in the changing habitat (49). This study demonstrates how SNP analyses can be used to identify functional domains of a protein as well as uncover a protein’s potential evolutionary history.

**Figure 7:**
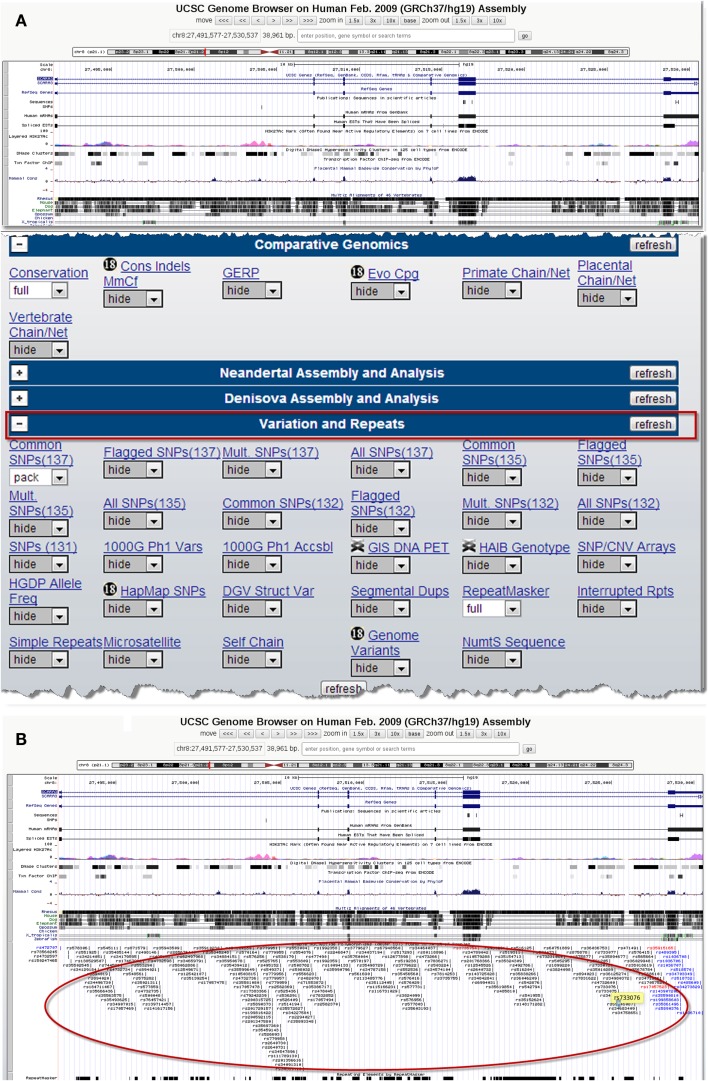
**Using the UCSC Genome Browser to search for single-nucleotide polymorphisms (SNPs) in SCARA3**. This browser contains multiple “tracks,” including the location of SNPs across the length of a protein. Here we show the output from inputting the NCBI RefSeq for SCARA3 isoform **(A)**. Further options to hide or show more annotation tracks are available directly below the graphical output. Under the “*Variation and Repeats*” tab, selecting “*pack*” under the “*Common SNPs*” option updates the output to include a full display of SNPs represented by their refSNP cluster ID numbers [**(B)**, circled]. Clicking on any of the refSNP cluster IDs leads to a link displaying further information regarding the SNP as well as a link to NCBI’s dbSNP database.

**Figure 8:**
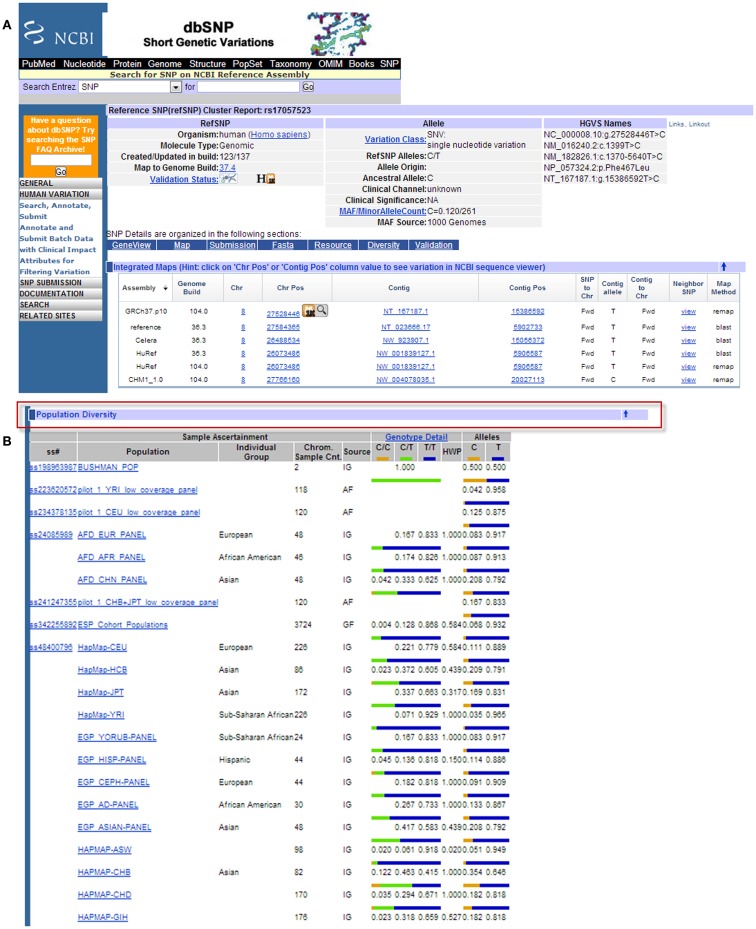
**Example results page from the NCBI dbSNP database for SCARA3 SNP rs17057523**. By following the link from the UCSC Genome Browser to dbSNP, more information is provided for SNP rs17057523 including allele frequencies, ancestral alleles, and chromosomal position **(A)**. Following this information on the database website, are other tabs that show more information regarding the SNP that may be useful to investigators. The “*Population Diversity*” section displays information regarding allele frequencies from different sampled populations **(B)**. Clicking any of the population links shows information on how the SNP was genotyped, the population sample size, and other experimental conditions used.

There are several publicly available online databases for the analysis of SNPs in a protein of interest (summarized in Table 5); here, we use The University of California, Santa Cruz (UCSC) Genome Browser^7^ to perform an analysis of SNPs present within SCARA3. Regions of interest can be searched for by entering the name of a gene or its corresponding chromosomal position. The Genome Browser contains multiple “tracks” that contain different types of annotation, including those based on NCBI RefSeqs, mRNA alignments, and UCSC Genes (50) (Figure 7). In addition, the browser can display reports regarding gene expression, regulation, and variation, among other information (50). The UCSC Genome Browser includes an annotated SNP track with over 23 million reference SNPs from NCBI’s SNP Database (dbSNP) (50) (Figure 7B). SNPs are annotated using a refSNP cluster ID number (rs#) which represents all SNPs, often from multiple population studies, that map to the same location in the gene. Additionally, each individual SNP within a cluster is associated with a SNP Accession number (ss#) (48). Selecting a refSNP cluster within the Genome Browser will display information such as the nucleotide change, chromosomal position, and type of variant as well as a link to the dbSNP database (Figure 8), which contains further detail on the population studies associated with the SNP, including observed allele frequencies and links to other resources such as GenBank and PubMed (48). The dbSNP database can also be accessed externally through NCBI, and individual SNPs can be searched for using their SNP Accession number, population study name, or via a BLAST search (51).

**Table 5:** **Publicly available single-nucleotide polymorphism (SNP) databases**.

Name	Hosted by	URL	Features	Availability	Reference
UCSC	University of California, Santa Cruz, CA, USA	http://genome.ucsc.edu/	Integrated browser displaying tracks built from annotation sets including SNPs, mRNA, disease association studies, and more	Web applet	Kent (68)
dbSNP	National Center for Biotechnology Information	http://ncbi.nlm.nih.gov/SNP/	Central database of SNPs with integrated data from multiple population studies including the 1000 genome project	Web applet	Sherry et al. (48)
GWAS central (formerly HGVbase database)	Institutes, Consortia, and individual laboratories	http://gwascentral.org/	Database of human genetic variation. Displays information on phenoytpes, genes, regions, or markers based on SNPs	Web applet	Fredman et al. (69)
ENSEMBL	European Bioinformatics Institute (EBI)	http://ensembl.org/	Contains available genomes of multiple species. Displays summary information regarding isoforms, SNPs, and other features of genes or proteins	Web applet	Flicek et al. (70)
HapMap	National Center for Biotechnology Information	http://hapmap.ncbi.nlm.nih.gov/	Contains integrated data of SNPs for haplotype analysis, finding tag SNPs, and for identifying GWAS hits	Web applet	Gibbs et al. (71)
1000 Genome Project	European Bioinformatics Institute	http://1000genomes.org	Contains 1092 available human genomes for analysis as well as summary documentation regarding SNPs and other variation	FTP download	Abecasis et al. (72)
HaploView	The Broad Institute	http://broadinstitute.org/	Calculates *r*^2^ and *D*′values for performing haplotype analysis of SNPs with HapMap data or user input data	For download on all major platforms	Barrett et al. (73)

*This list includes only SNP databases that focus on human and/or mouse sequences; other, more specialized databases may exist for other organisms. All databases listed accept novel SNPs from private and public organizations*.

When the UCSC Genome Browser is used to search for SCARA3, the resulting SNP track shows all of the reported SNPs within the gene (Figure 7B). Most of the annotated SNPs within SCARA3 are intronic variants, which would not alter the resultant protein; however intronic regions have been shown to be involved in regulatory processes. Of the three SNPs found in the exons of SCARA3, rs17057523 has the highest global minor allele frequency of 0.120 based on The 1000 Genome Project phase 1 data. Following the external link to dbSNP’s “*Population Diversity*” section shows that the SNP is found at higher frequencies in Asian populations, with allele frequencies up to 0.222 while other populations remain close to 0.1 (Figure 8). Additionally, the “*Multiz Alignment*” track shows areas of conservation between multiple vertebrates and suggests that SNP rs17057523 is present within a conserved area of SCARA3. Further testing by cloning the variant can help determine the function of this domain by examining functional differences between the SNP and wildtype allele.

## Further Analyses

What has been covered here represents the basic knowledge upon which most bioinformatic analyses will be conducted. As in any field, there are a plethora of examples of highly specialized bioinformatic tools and software that have been developed for the various sub-fields of immunology. For example, HLA peptide binding predictions can be made using various tools such as that available from the National Institute of Health^8^ (52). While an exhaustive list of such programs cannot be given, we suggest that the reader referred to other, more specialized reviews of such tools [(53–55) for example].

## Concluding Remarks

In our opinion, bioinformatics is a methodology that is under-utilized in immunological studies. Far from being inaccessible and complicated, many bioinformatic tools are straightforward and available via online servers, meaning that a researcher can obtain results instantaneously without fear of the often-steep learning curve associated with installable software. Although a strong background in computer science is an asset for more complicated techniques, in order to perform the analyses that we have described here, a passing familiarity with the cut and paste function is all that is required. If the reader is interested in going beyond this, there are excellent, freely available resources such as Software Carpentry^9^, Rosalind^10^, and online courses such as those available at Coursera^11^ and edX^12^. Acquiring vocabulary is probably the most challenging aspect of venturing into bioinformatics; however, one might argue that this is considerably easier to master than the language of immunology with its interminable number of interleukins, CD numbers, and signaling pathways. The goal of this review is to demonstrate some basic principles and techniques that are easily incorporated into the average bench scientist’s research and to encourage immunologists and cell biologists to consider using *in silico* approaches to generate and test hypotheses and answer research questions. Of course, like all hypotheses, those generated with *in silico* approaches must be experimentally tested. Whether *in silico* approaches are more or less accurate that traditional methods of hypothesis generation are yet to be evaluated. Our inquiry into the properties of SCARA3 indicates that these tools are immensely useful in generating hypotheses that can then be tested bench-side. Although many researchers have decried the lack of trained bioinformaticians and bioinformaticists, perhaps the best way to overcome the current shortage may be for scientists to become conversant in some of the basic techniques of bioinformatics in much the same way that we must be knowledgeable of the statistical tools required to analyze and understand our research.

## Conflict of Interest Statement

The authors declare that the research was conducted in the absence of any commercial or financial relationships that could be construed as a potential conflict of interest.

## Footnotes

^1^http://www.ebi.ac.uk/Tools/psa

^2^http://www.ebi.ac.uk/Tools/msa

^3^http://www.pdb.org

^4^http://www.ncbi.nlm.nih.gov/geo/

^5^https://www.ebi.ac.uk/gxa/

^6^www.immgen.org

^7^http://genome.ucsc.edu

^8^http://www-bimas.cit.nih.gov/index.shtml

^9^http://software-carpentry.org/

^10^http://rosalind.info/

^11^http://www.coursera.org

^12^http://www.edx.org
